# Systematic *in silico* Evaluation of *Leishmania* spp. Proteomes for Drug Discovery

**DOI:** 10.3389/fchem.2021.607139

**Published:** 2021-04-27

**Authors:** Crhisllane Rafaele dos Santos Vasconcelos, Antonio Mauro Rezende

**Affiliations:** ^1^Bioinformatics Plataform, Microbiology Department, Instituto Aggeu Magalhães, Recife, Brazil; ^2^Posgraduate Program in Genetics, Genetics Department, Universidade Federal de Pernambuco, Recife, Brazil

**Keywords:** drug repurposing, protein interaction network, drug targets, leishmaniasis, *Leishmania*

## Abstract

Leishmaniasis is a group of neglected infectious diseases, with approximately 1. 3 million new cases each year, for which the available therapies have serious limitations. Therefore, it is extremely important to apply efficient and low-cost methods capable of selecting the best therapeutic targets to speed up the development of new therapies against those diseases. Thus, we propose the use of integrated computational methods capable of evaluating the druggability of the predicted proteomes of *Leishmania braziliensis* and *Leishmania infantum*, species responsible for the different clinical manifestations of leishmaniasis in Brazil. The protein members of those proteomes were assessed based on their structural, chemical, and functional contexts applying methods that integrate data on molecular function, biological processes, subcellular localization, drug binding sites, druggability, and gene expression. These data were compared to those extracted from already known drug targets (BindingDB targets), which made it possible to evaluate *Leishmania* proteomes for their biological relevance and treatability. Through this methodology, we identified more than 100 proteins of each *Leishmania* species with druggability characteristics, and potential interaction with available drugs. Among those, 31 and 37 proteins of *L. braziliensis* and *L. infantum*, respectively, have never been tested as drug targets, and they have shown evidence of gene expression in the evolutionary stage of pharmacological interest. Also, some of those *Leishmania* targets showed an alignment similarity of <50% when compared to the human proteome, making these proteins pharmacologically attractive, as they present a reduced risk of side effects. The methodology used in this study also allowed the evaluation of opportunities for the repurposing of compounds as anti-leishmaniasis drugs, inferring potential interaction between *Leishmania* proteins and ~1,000 compounds, of which only 15 have already been tested as a treatment for leishmaniasis. Besides, a list of potential *Leishmania* targets to be tested using drugs described at BindingDB, such as the potential interaction of the DEAD box RNA helicase, TRYR, and PEPCK proteins with the Staurosporine compound, was made available to the public.

## Introduction

Leishmaniasis is a set of diseases caused by flagellated protozoa of the genus *Leishmania*, which can infect mammals since the Mesozoic era (Akhoundi et al., 2016). They are transmitted to humans and other mammals by the sting of phlebotomine of the genus *Phlebotomus* and *Lutzomyia*. Also, those parasites have a wide range of hosts, including canids, rodents, and humans (Maroli et al., 2013). Currently, with an estimated 1.3 million annual cases and 350 million people at risk of contracting the diseases, leishmaniasis is the cause of more than 20,000 deaths per year and remains a global public health problem (World Health Organization/Country Office for India, 2017; Gradoni, 2018).

World Health Organization (WHO) recommendations used to treat leishmaniasis are dependent on geographic region, as treatment failures are related to host genetics, their nutritional states, and to the selection of resistant strains and species present in certain regions. In general, the treatment involves pentavalent antimony, amphotericin B, paromomycin, pentamidine, and miltefosine, which are used in single drug or combined treatments. However, most of these drugs have unavoidable and sometimes irreversible side effects, such as pentamidine, which can cause insulin-dependent diabetes mellitus (World Health Organization, 2010; Nagle et al., 2014). The high incidence of these side effects has led to patients abandoning treatment or using the drugs incorrectly, causing the selection of resistant parasites (Ghorbani and Farhoudi, 2018). Drugs with milder side effects, such as miltefosine, have other downsides such as the long half-life that results in an extensive drug accumulation in the body and the teratogenic effects observed even when applied in dosages below the therapeutic dose (Dorlo et al., 2008, 2012; Rahman et al., 2017). In addition, other points that should be considered regarding the use of miltefosine as the main treatment are the high frequency of drug resistance, low availability and high cost, incompatible with poor endemic areas (Sunyoto et al., 2018). In this way, it is possible to understand the urgent need to apply methodologies capable of identifying new therapeutic targets and drugs with potential leishmanicide.

The process of discovering a new drug can be divided into a phenotypic screening or target-based approaches (Swinney, 2013). In phenotypic screening it is possible to select compounds with cytotoxicity against the whole cell, measure cellular function without imposing the need for prior knowledge about the targets; While in target-based approaches the process of developing a new drug has as its key point the discovery of a target or a set of targets (Gilbert, 2013; Schenone et al., 2013; Swinney, 2013). This knowledge about the target, different from what occurs in the phenotypic screening method, allows access to crucial information for the optimization of compounds, such as knowledge about the target/drug binding site (Gilbert, 2013; Li and Kang, 2017). However, target selection is often based on biological evidence which results in the analysis of a small target group, exploring only a small portion of the total proteome and neglecting potentially therapeutically important new drug targets (Patel et al., 2013; Schenone et al., 2013).

Thus, when the goal is to select new targets for drug development, where large-scale analyzes of molecules and compounds are performed, computational methods are presented as an initial choice, providing methodologies capable of identify targets from a proteome starting from the recognition of patterns present in compounds and their already known targets (Schenone et al., 2013), being a powerful methodology for integrating data, accelerating the first stages of the study, and reducing the cost of the process (Sanseau et al., 2012; Patel et al., 2013; Schenone et al., 2013). The detection of potential pharmacological targets by computational methods can be focused on the target or the ligand using data extracted from sources such as protein interaction network, sequence or structural similarity of proteins against approved drug targets, the similarity between structures of the ligands and their side effects, druggability regarding protein structure and gene expression (Sliwoski et al., 2014). However, to exploit the potential of such data, the integration of all available information must be carried out effectively.

In this context, we applied computational analyses integrating information in biological and pharmacological contexts to infer interactions between the proteome of *Leishmania infantum* and *Leishmania braziliensis* with available drugs (BindingDB compounds). These species are responsible for the different clinical manifestations of leishmaniasis in Brazil, a country that is among the global regions with the highest numbers of cases (Ministério Da Saúde, 2006; Brasil et al., 2017, 2019; Pan American Health Organization, 2018). Thus, the possibility of drug repurposing could be verified and new information about the druggable proteome of *Leishmania* species was recovered.

## Materials and Methods

### Data

*L. braziliensis* and *L. infantum* predicted protein sequences were obtained from the TriTrypDB database (release 9.0) (Aslett et al., 2010), while the protein tertiary structures, and data regarding protein interactions for each above proteome, were retrieved from a database created in a previous work of our group (dos Santos Vasconcelos et al., 2018).

Data about known interactions between proteins without restricting by any specific organism and drugs were obtained from the BindingDB database (Liu et al., 2007) on September 5, 2019. This repository was chosen as it integrates interaction data from several public databases and assigns an affinity value for a pair of proteins and drugs. With protein IDs available at BindingDB, it was possible to obtain the aminoacid sequences and three-dimensional structures of each protein target using the Uniprot database (Bairoch et al., 2005) and *Protein Data Bank* (PDB) (Berman et al., 2000), respectively. Proteins without X-ray crystallography three-dimensional structures at PDB had their three-dimensional models retrieved from the SwissModel repository (Bienert et al., 2017), which uses automated comparative modeling techniques to predict protein structures. All PDB format files were processed to remove ligands. This was necessary once a protein can not have a bound ligand when it is used as input to tools that perform searches by regions (pockets) able to interact with drugs. Therefore, to identify pockets, the structures must be free of ligands.

### Construction of Similarity Matrices

To evaluate the proteins from Binding DB regarding their chemical (the physico-chemical properties of three-dimensional structure) and biological (molecular function, biological processes in which the protein is involved and its subcellular location) contexts, a methodology similar to that described by Patel et al. was used, which consists of classifying potential targets based on the annotation of characteristics about the three-dimensional structure, druggability, functional class and subcellular location (Patel et al., 2013). However, the methodology applied here proposes a direct comparison of each characteristics annotated for a proteome with the same characteristics extracted from already known drug targets. Thus, features from structural, functional, and sequence levels were extracted from the recovered targets. The same features were extracted for the *Leishmania* species proteomes to compare them to targets recovered from BindingDB, and thus to build similarity matrices between described targets and *Leishmania* proteins.

Therefore, considering 2 proteins *t*_*i*_ and *p*_*j*_: *t*_*i*_ is a protein described as a reference drug target from Binding DB; and *p*_*j*_is a protein belonging to the *L. braziliensis* or *L. infantum* proteomes. We constructed 5 similarity matrices *S*^*x*^ where *x* indicates the information used to construct the matrix, and *S*_*ij*_ represents the similarity relationship between *t*_*i*_ and *p*_*j*_. Hence, similarity matrices were built considering the similarity between the binding sites of drugs (*S*^*b*^), the druggability value between the most similar binding sites (*S*^*d*^), the subcellular location (*S*^*s*^), the biological processes that proteins were involved (*S*^*p*^), and the molecular functions of the proteins (*S*^*m*^).

The first two matrices (*S*^*b*^ and *S*^*d*^) were built using the information on ligandability at the protein structural context. Ligandability is the ability of a protein to bind with high affinity to small drug-like compounds (Hussein et al., 2016). The ligandability prediction using the three-dimensional structure is performed by evaluating physicochemical parameters such as polar and non-polar surface area, surface complexity, and pocket dimensions (drug binding site) (Le Guilloux et al., 2009; Volkamer et al., 2010). Thus, to produce both matrices cited above, the BindingDB target structures and the *L*. *braziliensis* and *L. infantum* protein structures were submitted to the binding site detection algorithm present at the Fpocket tool (Le Guilloux et al., 2009). This software was chosen because the results obtained are more accurate when compared to other tools, such as SiteFinder, PocketFinder, and SiteMap (Le Guilloux et al., 2009; Schmidtke et al., 2010). With the identification of each binding site, a druggability index/score is assigned to each binding site found in the protein structures (scores values ranging from 0 to 1, with 0 assigned to pockets with a low probability of binding to a drug-like molecule, and 1 assigned to pockets with a high probability). Also, a threshold (druggability score ≥ 0.5) established by the Fpocket tool was applied to ensure the druggability of the binding sites of evaluated targets. Thus, the structure of the binding sites and their druggability index was obtained.

The predicted binding sites of each *Leishmania* spp. protein was then compared to binding sites of BindingDB protein targets using the Pocketmatch tool version 2.1 (Yeturu and Chandra, 2008). In this way, all predicted binding sites of a *p*_*j*_ protein were aligned with the binding sites of the *t*_*i*_ protein, generating similarity values. The highest similarity value is the one selected to $S_{ij}^{b}$.

In order to construct the similarity matrix $S_{\;}^{d}$, the druggability values for the most similar binding sites between proteins *p*_*j*_ and *t*_*i*_ were considered. Thus, the $S_{ij}^{d}$ of this matrix was calculated using the following equation:

$$\begin{array}{l} S_{ij}^{d}=1-\;D_{ij} \end{array}$$

being *D*_*ij*_ the one-dimension Euclidean distance between the *t*_*i*_ and *p*_*j*_ druggability values.

The matrices referring to Molecular Functions and Biological Processes were built using semantic similarity between Gene Ontology (GO) terms. The GO terms of each BindingDB protein target were retrieved from the Uniprot database. For those without GO annotation, as well as for the proteomes of *L. braziliensis* and *L. infantum*, the Blast2GO tool (Conesa and Götz, 2008) was used to assign GO terms. Blast2GO is a tool for automatic functional annotation based on homology transfer (Conesa and Götz, 2008). The terms retrieved from Uniprot, as well as those annotated by Blast2GO, were submitted to the *csbl.go* R package (Ovaska et al., 2008) to calculate semantic similarity between proteins *p*_*j*_ and *t*_*i*_ applying the Relevance algorithm (Schlicker et al., 2006). Hence, two matrices were produced, one containing the similarity between the terms referring to Molecular Function (*S*^*m*^), and the other containing the similarity between the Biological Process terms (*S*^*p*^).

The subcellular location (SL) matrix consisted of a binary matrix *S*^*s*^ for a pair of *t*_*i*_ *and p*_*j*_ proteins, being $S_{ij}^{s}=1$ when the pair of proteins shared the same subcellular location, and $S_{ij}^{s}=0$ when the proteins had different subcellular locations. The data about the subcellular location were retrieved from the Uniprot database. For BindingDB protein targets and *L. braziliensis* and *L. infantum* proteins without that information, the WolfPsort tool (Horton et al., 2007) was applied to predict the subcellular localization.

### Selection and Evaluation of Potential Drug Targets

The members of *Leishmania* proteomes were classified according to the degree of similarity between them and BindingDB drug targets using as basis the constructed similarity matrices. Thus, the greater the similarity of a *Leishmania* protein to a BindingDB drug target, the greater the probability of that protein being a compound target. In order to consider only the proteins most likely to interact with a drug, the following criteria were used: 1 - a *Leishmania* protein must have a binding site and its druggability similar to the same BindingDB drug targe; 2 - a protein that passes the previous criterion must also be similar to the same BindingDB drug target in at least two of the other three metrics evaluated, being Molecular Function, Biological Process, or Subcellular Location. Thus, it is possible to infer that the selected *Leishmania* protein may have the potential to bind to the same compounds that interact with the BindingDB drug target.

However, it is important to remember that even if a protein has the ability to interact with a drug, for that protein to be considered truly druggable, its interaction with a drug must generate a directed effect on the organism. Thus, a protein is potentially interesting as a drug target when the maintenance of its function is essential to keep the organism survival (Schmidtke and Barril, 2010; Desaphy et al., 2012; Hussein et al., 2016). Thus, to evaluate the essentiality of the *Leishmania* species proteins, we considered their positions in the network of protein interaction.

*L. braziliensis* and *L. infantum* proteins had their essentiality evaluated through their positioning in protein interaction networks (PIN). Interaction data were retrieved from previous work of our group (Rezende et al., 2012; dos Santos Vasconcelos et al., 2018) integrated with interactions data retrieved from the String database (Szklarczyk et al., 2015). To analyze the essentiality of a protein, the Cytoscape tool version 3.8.0 (Shannon et al., 2003) was used to calculate the centrality indexes: Degree Centrality, Neighborhood Connectivity, and Betweenness Centrality. These metrics reflect the centrality-lethality rule, which describes high-degree proteins as essential and can be used as good predictors of biological importance (Zotenko et al., 2008).

Due to the existence of two main distinct evolutionary stages in the biological cycle of the *Leishmania* species, and only one of these stages parasitizing humans (Bates and Rogers, 2004), the levels of gene expression between the two stages were evaluated. Thus, RNA sequencing data from *L. braziliensis* species (PRJNA494068) were retrieved from the Sequence Read Archive database (SRA) (Kodama et al., 2012), mapped and aligned with reference sequences through the Bowtie2 tool (Langmead and Salzberg, 2012). The data were then formatted using the SAMtools tool (Li et al., 2009), and the reads aligned for each gene were counted using the Htseq-count tool (Anders et al., 2015). The expression analysis between the evolutionary stages was performed using the DESeq2 library (Anders and Huber, 2010) of the R programming environment. Thus, allowing the identification of genes that are expressed in the amastigote evolutionary stage.

In order to reduce the chances of side effects, we carried out an analysis of the similarity between *Leishsmana* ssp. proteins selected previously and the human proteome (recovered from the Uniprot database), using the alignment tool Blastp. We used a cut-off value of 50% similarity based on the analysis of Patel et al. which showed a relationship between structure, function, and sequence for sequence similarity above 50% (Patel et al., 2013). Considering similar proteins interact with similar drugs, evaluating the similarity with human proteins may reflect the possibility of side effects (Zhou et al., 2015). All data retrieved and produced during this study were stored using a relational database model through the MySQL database manager tool. The completed database scheme and all the data produced are available as Supplementary Material on the https://leishtargets.github.io/ in order to facilitate the reproducibility of this research and data retrieval to guide other projects.

## Results

### Data

Our last access to BindingDB contained 64,025 interactions described with high-affinity value (better than 10 uM) between 3,128 proteins and 29,353 compounds. The choice for interactions with purchasable compounds was made due to the facility of their acquisition for subsequent projects with biological tests. *L. braziliensis* and *L. infantum* proteomes addressed in this study contained a total of 8,357 and 8,239 proteins, respectively. None of these proteins were found in the BindingDB database as targets with high-affinity value for a purchasable compound, and only two BindingDB protein targets belonged to the same genus addressed in this study, and they are Bifunctional dihydrofolate reductase-thymidylate synthase (LmjF.06.0860) and Pteridine reductase 1 (LmjF.23.0270), both proteins of *Leishmania major*. However, there are other proteins of the *Leishmania* genus described as interacting with compounds in BindingDB. These proteins were not recovered due to a low-affinity value with the tested compounds or due to the impossibility of buying such compound. Thus, we identified 6 proteins of the genus *Leishmania* that were tested for 40 compounds: Farnesyl pyrophosphate synthase (LmjF.22.1360); Glyceraldehyde-3-phosphate dehydrogenase-like protein (LmxM.34.4750); NAD-dependent protein deacetylase (LinJ.26.0200), Putative lanosterol 14-alpha-demethylase (LinJ.11.1100), N(1), N(8)-bis(glutathionyl)spermidine reductase (LinJ.05.0350) and Tubulin alpha chain (Q1A5Y2). Twenty-five of those compounds are present in our similarity data due to their high-affinity with other BindingDB protein targets (Supplementary Material 1).

### Construction of Similarity Matrices

#### Druggability of Binding Sites

For the analyses that involved the identification of protein binding sites, we recovered a total of 2,641 BindingDB protein targets with structure, of which 1,592 and 1,049 were from the PDB and SwissModel, respectively. Among the BindingDB targets recovered, 14 structures predicted by SwissModel suite had <80% coverage and/or identity when compared to the protein sequence deposited in the Uniprot; 6 protein structures (3j9m, 4u4r, 5gjr, 5j4z, 5vfl, 6ek0) could not be recovered from the PDB in an automated way due to the size of the deposited structure. In the end, 468 BindingDB proteins had no structure deposited in both repositories used.

After treatment, the atomic coordinate files (PDB files) were submitted to the Fpocket tool to predict binding sites and assign a score. We were unable to identify binding sites for 17 BindingDB protein targets. Thus, further analyses were performed only to coordinate files of the binding sites that were able of being evaluated by the PocketMatch tool. Therefore, 2,057 BindingDB protein targets displayed binding sites according to these criteria.

The total protein structures of *L. braziliensis* and *L. infantum* proteomes were 681 and 708, respectively (dos Santos Vasconcelos et al., 2018). The three-dimensional conformations produced through comparative modeling did not need to be processed to remove ligands, and all recovered structures were submitted to the Fpocket tool. The Fpocket tool was unable to recover binding sites for 12 and 7 proteins of *L. braziliensis* and *L. infantum*, respectively. Afterward, the atomic coordinates related to the predicted binding sites of *L. braziliensis* and *L. infantum* proteins were compared to the binding sites of BindingDB target proteins. That comparison was performed using the PocketMatch tool.

Therefore, matrices that depend on three-dimensional structure (*S*^*b*^ and *S*^*d*^) were composed of 2,057 BindingDB protein targets and 669 and 701 proteins of *L. baziliensis* and *L. infantum*, respectively. Based on the analysis carried out with the Fpocket, it was possible to identify that almost 99% of the protein structures of the two *Leishmania* species had binding sites that could be identified by Fpocket, with a druggability score between 0.021 and 0.926 for *L. braziliensis* and between 0.042 and 0.985 for *L. infantum* (see Table 1 and Supplementary Material 2). Considering the threshold indicated in the tool documentation, which reports a druggability score >0.5 for druggable proteins, we identified that almost 88% of the protein structures of both *Leishmania* species had druggable binding sites.

**Table 1:** Total druggable binding sites.

**Species**	**Predicted structures**	**Protein structures with binding sites**	**Protein structures with druggable binding sites**
*L. braziliensis*	681	669 (98.2%)	599 (87.9%)
*L. infantum*	708	701 (99%)	629 (88.8%)

When we evaluated the druggability similarity matrices and binding sites between BindingDB drug targets and *L. braziliensis* and *L. infantum* proteins, only 277 and 265 proteins of *L. braziliensis* and *L. infantum*, respectively, showed similar structure of their binding sites ($S_{ij}^{b}\geq 0.7$). When we integrated binding sites with druggability similarity, it was possible to identify the number of *Leishmania* proteins with the structure of binding sites and druggability scores similar to the same BindingDB drug target, essential information to infer the drug repurposing to a new protein. In this way, we were able to identify that 192 proteins of *L. braziliensis* and 210 proteins of *L. infantum* have binding sites similar to known drug targets $\left(S_{ij}^{d}\;\geq 0.8\;and\;S_{ij}^{b}\geq 0.7\right)$, considering both their three-dimensional conformation with their chemical composition, and their druggability score (data on the similarity of druggability and binding sites can be retrieved from the Supplementary Material at https://leishtargets.github.io/).

#### Gene Ontology Terms and Subcellular Location

When we analyzed the GO annotations, most of the recovered BindingDB protein targets were involved in metabolism processes, cell communication, and development. When verifying the enrichment of the terms using the David tool version 6.8, it was possible to see that the BindingDB proteins were enriched for protein serine/threonine kinase activity, protein phosphorylation, protein kinase activity, and ATP binding (Enrichment Score: 175.78). The analysis performed with the *csbl.go* package allowed us to identify significant similarity (Relevance similarity ≥ 0.8) between BindingDB protein drug targets and approximately 92% of proteins from both *Leishmania* species proteomes (7,625 and 7,525 proteins of *L. braziliensis* and *L. infantum*, respectively), considering the GO terms related to Biological Processes. When evaluating this similarity using the matrices for GO terms related to Molecular Functions, we identified that near 82% of both *Leishmania* proteomes had similarities with BindingDB proteins (6,905 and 6,815 proteins of *L. braziliensis* and *L. infantum*, respectively).

Based on the data predicted by the WolfpsortII tool, it was possible to identify the majority (32.8%) of the BindingDB recovered drug targets have predicted location for the plasma membrane, while 22.7, 16.17, and 14.19% were related to the cytosol, nuclear, and extracellular locations, respectively. There were also predictions for chloroplast, cytoskeleton, endoplasmic reticulum, Golgi apparatus, lysosome, mitochondria, peroxisome, and vacuolar membrane, with ~3.4% of proteins predicted for 2 different subcellular locations.

It was possible to predict subcellular location for most proteins of *L. braziliensis* and *L. infantum*. Only the proteins LbrM.03.0710 and LbrM.09.0840 of *L. braziliensis* and LinJ.15.1300 of *L. infantum* did not return any prediction on subcellular location. All these proteins are described in the Uniprot database as uncharacterized proteins. We found that approximately 34% of both proteomes had subcellular location predicted as nuclear, while 18, 16, and 14% had predicted location as cytosol, mitochondria, and plasma membrane, respectively. There were also predictions for cytoskeleton, endoplasmic reticulum, extracellular, Golgi apparatus, lysosome, and peroxisome, with ~5% of both proteomes predicted for 2 different subcellular locations. When evaluating the similarity matrices, we verified all proteins of both *Leishmania* proteomes had subcellular localization similar to some drug targets.

### Selection of Potential *Leishmania* Drug Targets

**Figure 1:**
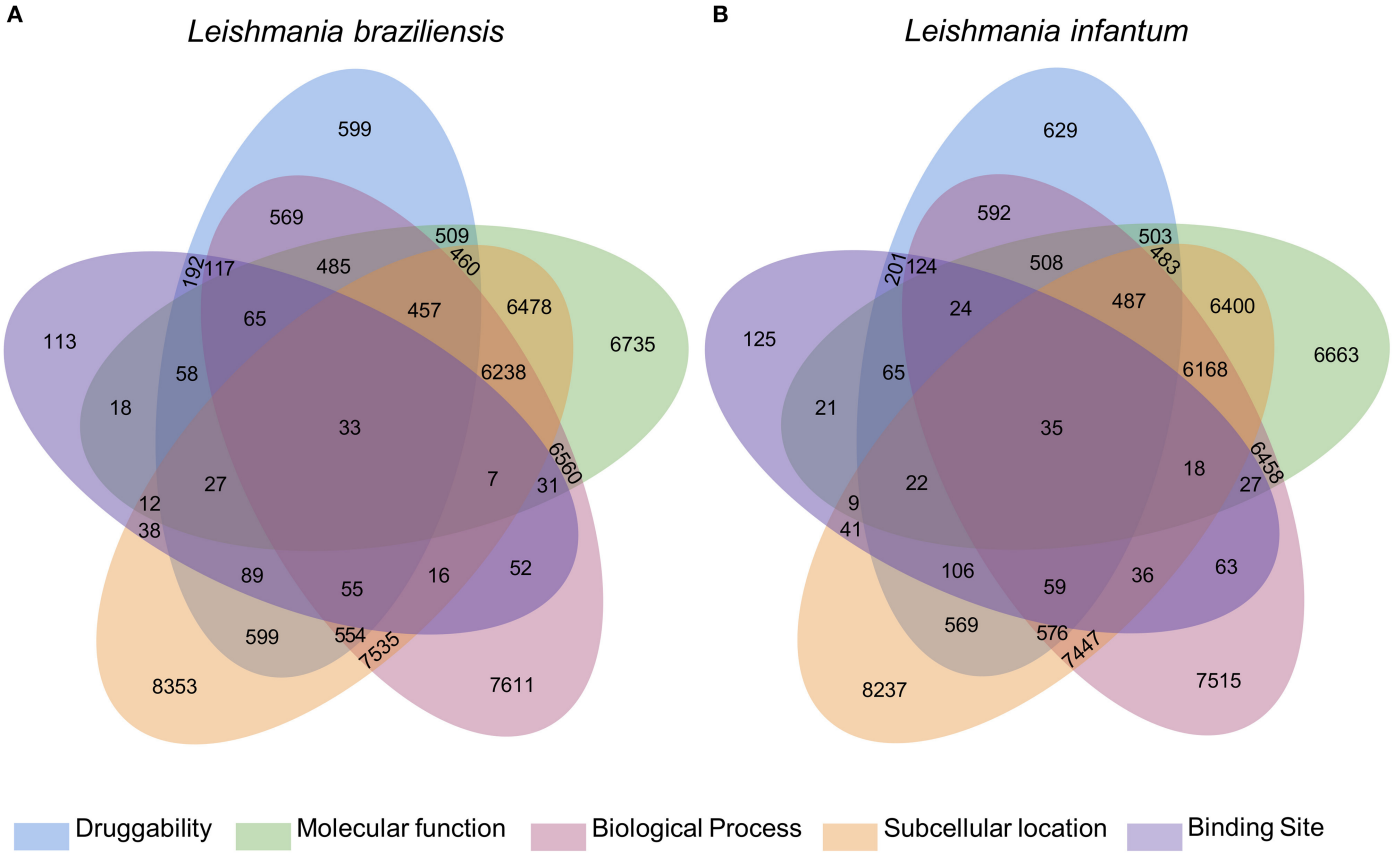
Diagram with results of the multidisciplinary approach to select targeting drugs from *Leishmania* species proteome. The Venn diagram illustrates the integration of the results found for the selection of druggable proteins based on structural, functional, and subcellular location evidence. From these results, it is possible to find 119 and 116 proteins of *L. braziliensis* **(A)** and *L. infantum* **(B)**, respectively, with evidence of potential drug targets for having similarity with known targets in at least 4 methodologies used, 2 of which are based on the formation and composition of the target binding site (druggability and binding site).

The integration of all similarity data was used to select the most pharmacologically interesting proteins (Figure 1). Thus, we identified that 119 and 116 *L. braziliensis* and *L. infantum* proteins, respectively, were similar to BindingDB drug targets in at least 4 levels of similarity and that 33 and 35 of those proteins were similar to drug targets in 5 levels of similarity. We considered these last set of proteins the most promising to interact with drugs to be redirected (Supplementary Materials 3, 4).

These results indicate which *L. braziliensis* and *L. infantum* proteins present evidence of potential drug targets based on structural, functional, and subcellular location data. However, it was necessary to verify which of these proteins are present in the evolutionary stage of pharmacological interest, and which of these proteins would have a significant impact on the physiological stability of the organism, if used as a drug target.

### Evaluation of Promising *Leishmania* Drug Targets

To analyze the presence of *Leishmania* proteins similar to drug targets at the evolutionary stage of amastigote, we analyzed the gene expression on data retrieved from SRA databases. To date, only one study deposited in the SRA (PRJNA494068) had total RNA sequencing data for the two distinct evolutionary stages (amastigote and promastigote) of wild strain of *L. braziliensis* species with no chemical interference. There was no data following such criteria for *L. infantum*. The results of expression analysis for *L. braziliensis* were used to infer gene expression for orthologs in *L. infantum*. Thus, to identify expressed genes in the evolutionary form present in the human host, the general distribution of the genes was analyzed, according to the log2 fold change between amastigote and promastigote forms. A total of 3,506 expressed genes were positively regulated with absolute values of log2 fold change > 0 and adjusted *p*-value ≤ 0.05 (Supplementary Material 1).

**Figure 2:**
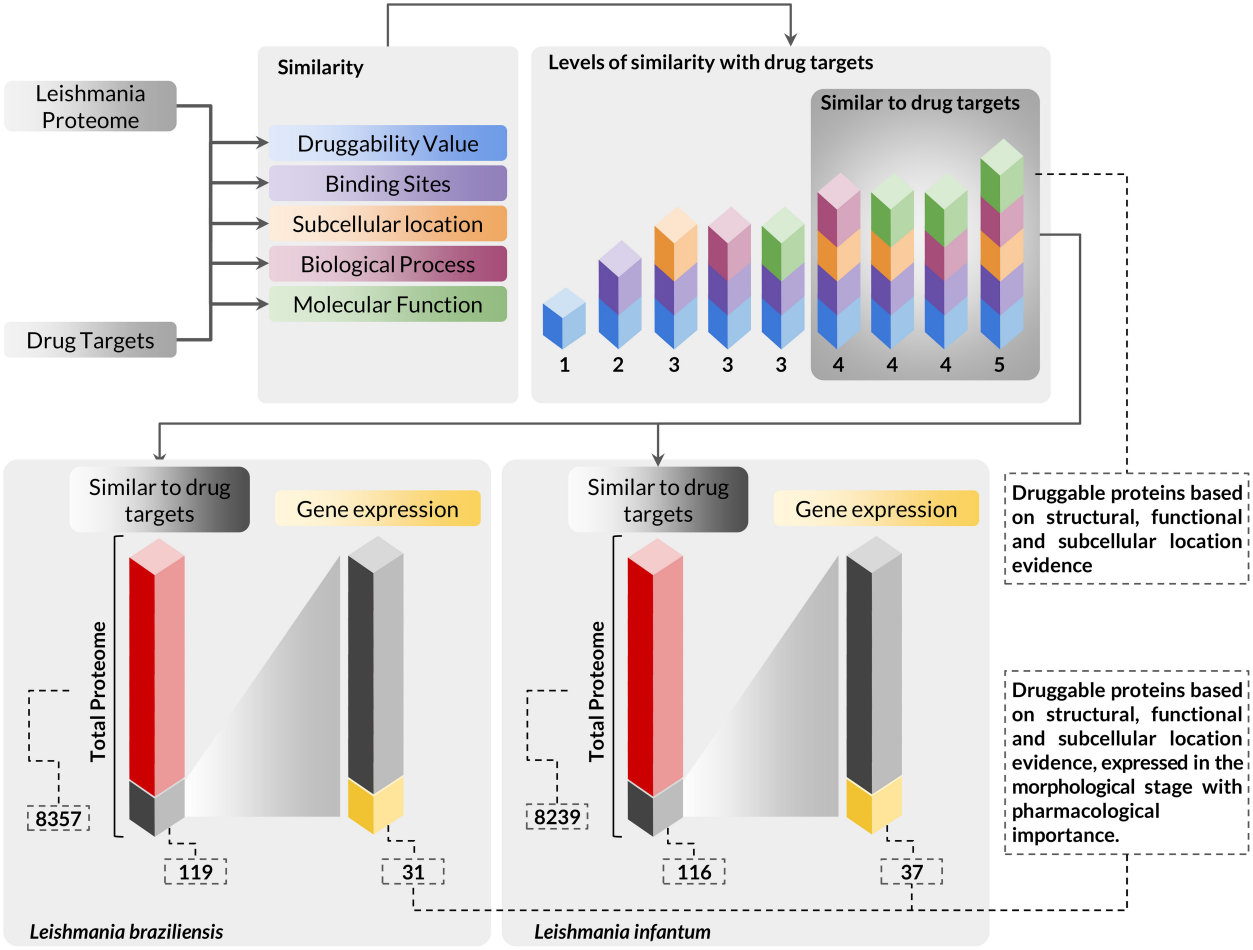
Workflow presenting the scheme and criteria used for the selection of druggable proteins of pharmacological interest in *Leishmania* sp. proteomes. The complete proteome of *Leishmania braziliensis* and *Leishmania infantum* was submitted to analysis using a multidisciplinary methodology for the selection of druggable proteins based on structural, functional, and subcellular location evidence, thus identifying 119 proteins in *L. braziliensis* and 116 proteins in *L. infantum* with druggable protein criteria. The proteins selected in this context were also evaluated for the differential expression of their respective genes to verify the presence of this protein in the evolutionary stage of pharmacological interest. In this analysis, we identified that among the druggable proteins, 31 and 37 proteins of *L. braziliensis* and *L. infantum* (Supplementary Materials 5, 6), respectively, had their genes positively regulated in the evolutionary stage of interest.

Integrating the results of expressed genes with the evidence for druggable targets, we identified that 31 and 37 proteins of *L. braziliensis* and *L. infantum*, respectively, had evidence of druggability and evidence of expression of its genes in amastigote, the evolutionary stage of pharmacological interest (Figure 2) (Supplementary Materials 5, 6). We evaluated the functional annotation of potential drug targets in *Leishmania* spp. proteomes using the categorization of GO terms related to biological process and molecular function (Supplementary Material 1). From these analyses, we found that the selected targets are strongly related to metabolism, cell organization, and biogenesis, and have molecular function mainly associated with protein binding and catalytic activity.

**Figure 3:**
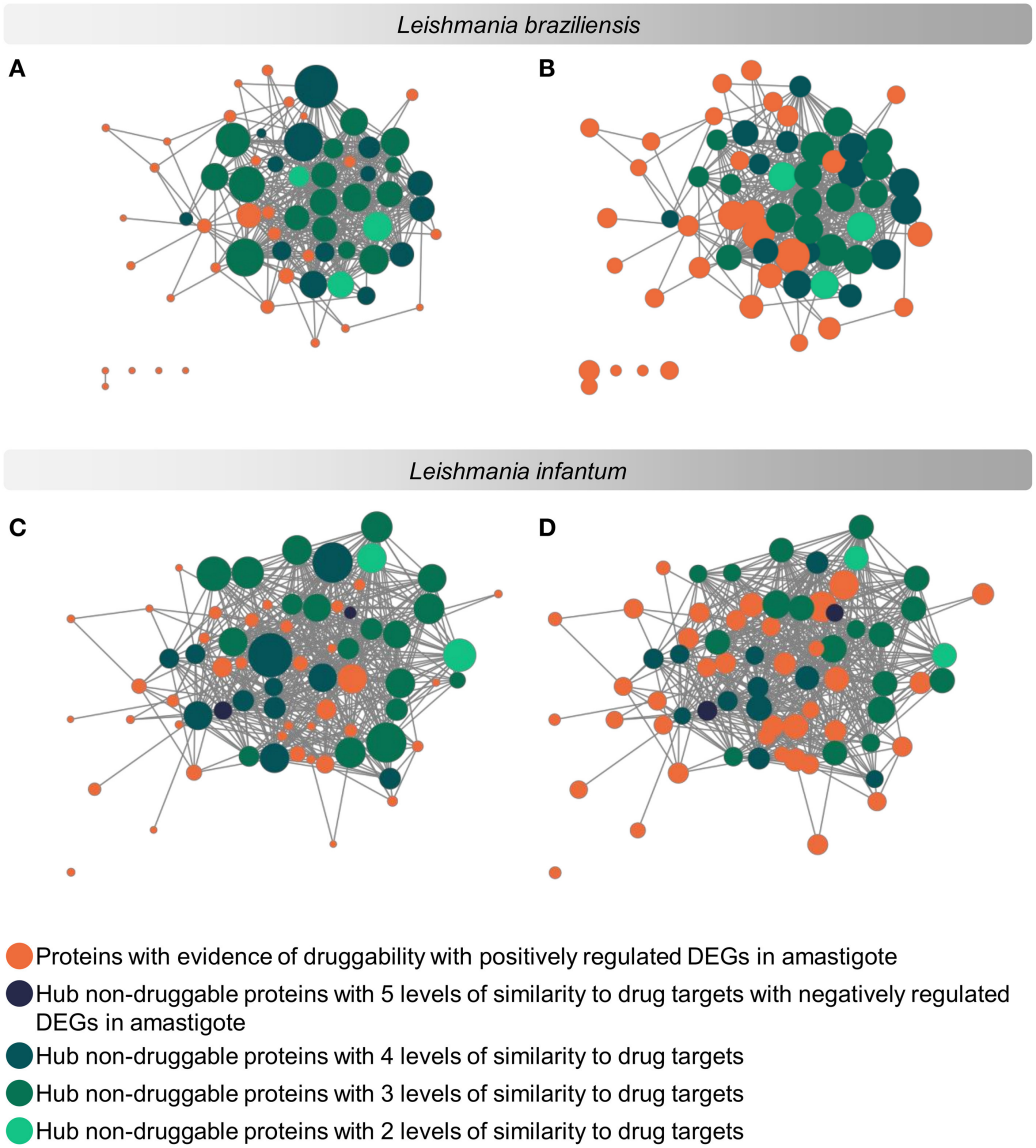
Subnetwork of protein interaction for *Leishmania* sp. focused on potential drug targets and hub proteins. The complete interaction network (Supplementary Materials 7, 8) was obtained from the union of protein interaction data produced in previous works of the group with the data deposited in the String database. The subnetworks were generated by selecting the hub proteins for degree centrality, hub proteins for centrality, and the potential drug targets. **(A,C)**: Size of the nodes reflect the Degree Centrality. **(B,D)**: The size of the nodes reflects the average connectivity of neighbors of a given node (Neighborhood Connectivity). The networks were noted according to the levels of similarity with known drug targets.

The evaluation regarding the importance of each parasite protein for the physiological stability of the organism was analyzed considering the degree of interaction of each protein in a complex of protein interaction networks (PINs). The networks used contained 3,397 proteins of *L. braziliensis* and 3,254 proteins of *L. infantum* (Supplementary Materials 7, 8 and data on protein interaction network can be retrieved from the Supplementary Material at https://leishtargets.github.io/). We identified that among the 31 proteins of *L. braziliensis* with evidence of druggability, 7 proteins had <20 interactions, while 7 proteins had more than 100 interactions. Considering the 37 proteins of *L. infantum*, we identified that 5 proteins had <20 interactions, while 9 proteins had more than 100 interactions (Figure 3).

**Figure 4:**
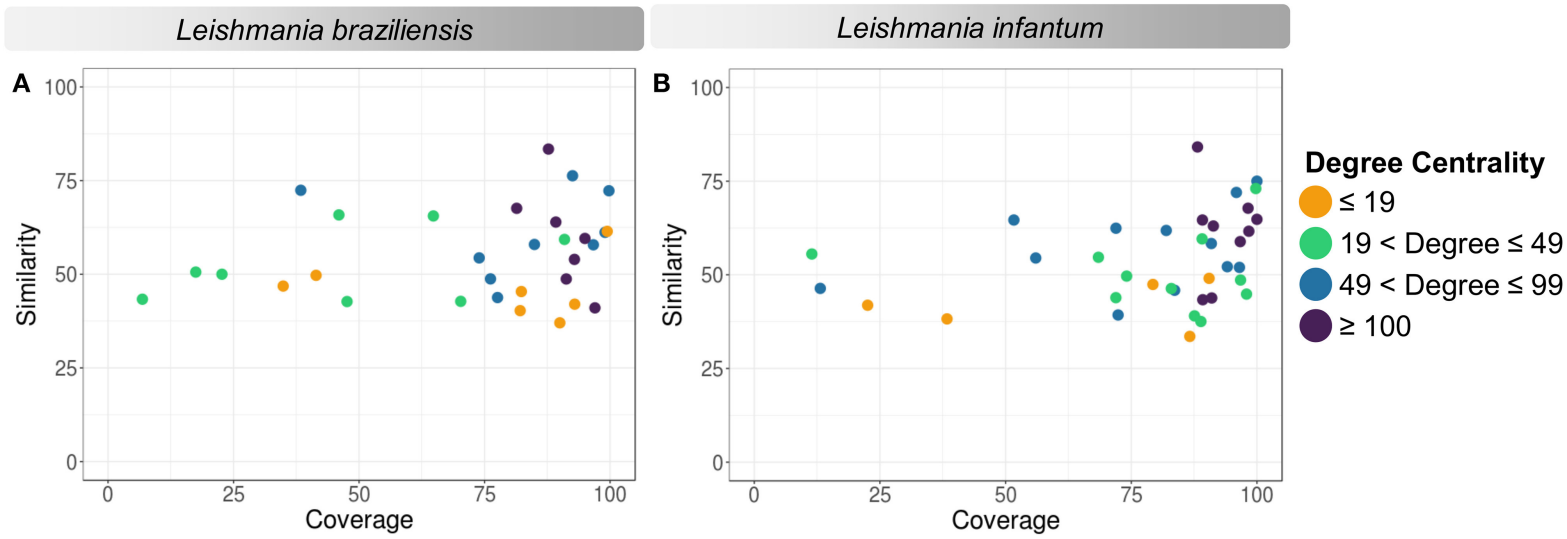
Degree centrality, similarity, and coverage against human proteome of potential target proteins of *Leishmania* sp. **(A)** *Leishmania braziliensis*; **(B)** *Leishmania infantum*. The degree centrality of each protein is reflected in orange (degree ≤ 19), green (19 < degree ≤ 49), blue (49 < degree ≤ 99), and purple (100 ≤ degree).

**Figure 5:**
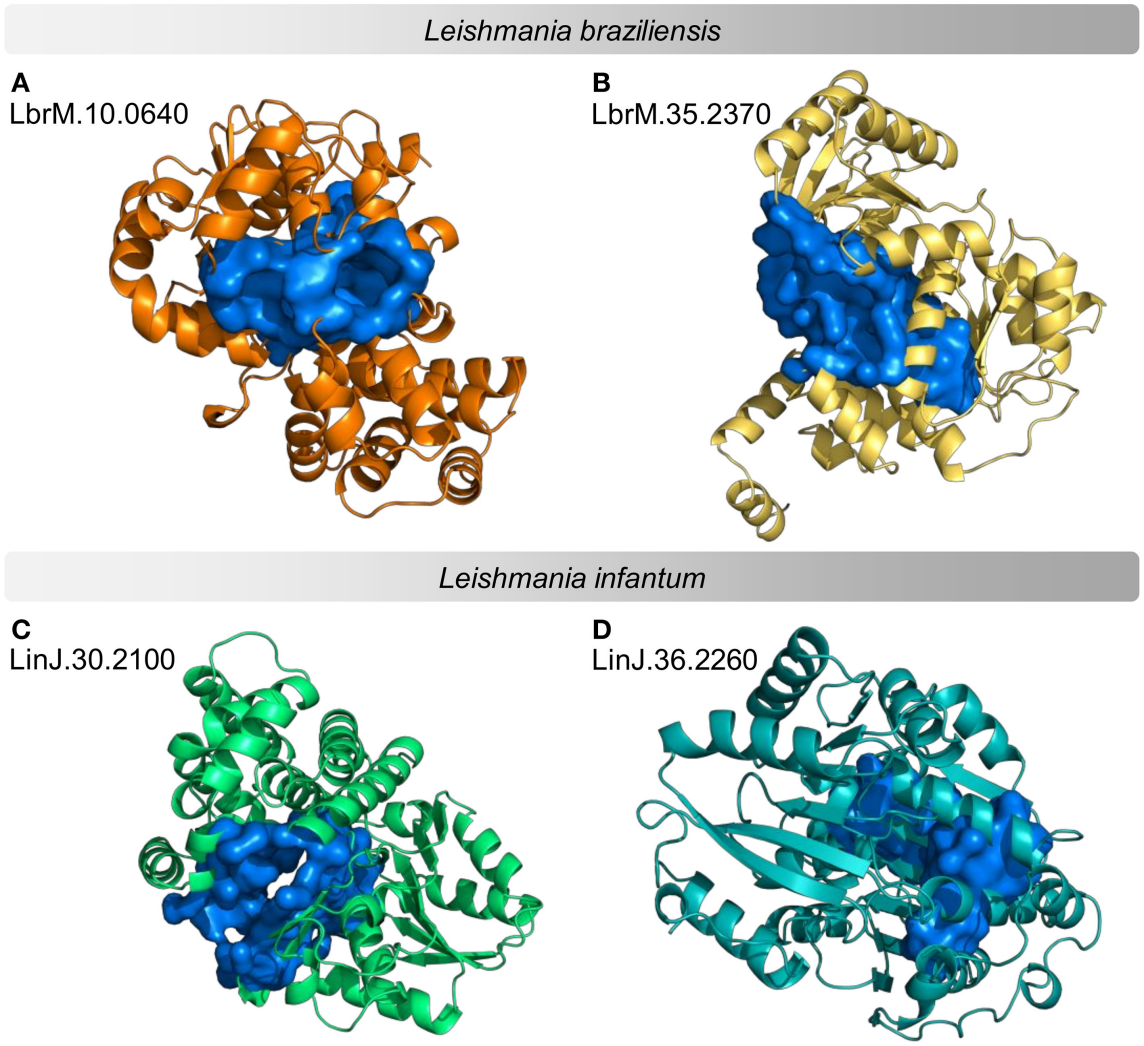
Example of predicted structures and druggable binding sites for potential protein targets in proteomes of *Leishmania* sp. Predicted three-dimensional conformations associated with a druggable binding site for 4 examples of potential drug targets. **(A,B)** examples of *L. braziliensis* proteins **(C,D)** examples of *L. infantum* proteins. The structures on the left are examples of druggable proteins with <50% similarity to the human proteome. The structures on the right are examples of druggable proteins with the highest Neighborhood Connectivity value in the subnetwork.

According to the PINs, the potential selected targets are not the main hubs proteins in the network, however, they tend to be directly connected or to the main hub proteins. It was still possible to notice that the network hubs mostly have a high similarity with drug targets, but they were not within the criteria established for potential drug repurposing. Thus, the network's hubs can be described as potential targets for the development of new drugs. When evaluating networks using the average connectivity of neighbors, we observed that 2 potential selected targets for each proteome, putative DEAD box RNA helicase (LbrM.35.2040, LbrM.35.2370, and LinJ.36.2260) and putative ATP-dependent RNA helicase (LinJ.28.1420) were present in highly connected neighborhoods, having a neighborhood connectivity value superior to hub proteins (with many direct interactions), thus being important for coefficient clustering and consequently important for the stability of the protein networks (Fox et al., 2011). The evaluation of similarity with the human proteome was carried out in an integrated way with the degree centrality (Figure 4), in order to weigh the importance for stability in parasite physiology and the possibility of side effects. Based on this analysis, the potential targets with a higher degree of centrality also had a greater similarity with the human proteome, especially when considering the coverage between the proteins. Just as proteins with lower degree centrality also have less similarity, data consistent with one of the first characteristics found in biological interaction networks, which is the conservation between highly connected protein species (Sun and Kim, 2011). However, it is possible to identify 4 and 5 proteins in *L. braziliensis* and *L. infantum*, respectively (Table 2), with degree centrality >50 and similarity against the human proteome <50% (Figure 4). The evaluation of these characteristics makes these proteins with the greatest potential to be tested as drug targets since they have the availability of a three-dimensional conformation that can be exploited for optimization of compounds (Figure 5), they have evidence of druggability with the similarity of the binding site and druggability score with known targets, in addition to having a similar molecular function and biological process, and share the same subcellular location.

**Table 2:** Potential drug targets with relative PIN essentiality and <50% similarity to the human proteome.

**Protein**	**Description**	Similarity^*^	Coverage^*^	**Number of potential compounds to interact with Leishmania proteins**	**Number of potential compounds already tested for Leishmania deposited in BindingDB**
LbrM.10.0640	Glycerol-3-phosphate dehydrogenase (GPHD)	48.7	91.2	63	8
LbrM.29.2360	Putative kinesin	48.7	76.1	640	0
LbrM.30.2040	Putative alcohol dehydrogenase	41.0	72.2	35	0
LbrM.35.6570	Putative small G-protein	43.7	77.5	341	1
LinJ.02.0710	Hypothetical Protein - Dipeptylcarboxypeptidase	45.8	83.6	42	0
LinJ.10.0560	Glycerol-3-phosphate dehydrogenase (GPHD)	43.8	91.0	52	0
LinJ.27.1710	Phosphoenolpyruvate carboxykinase	46.3	13.1	22	0
LinJ.30.2100	Putative alcohol dehydrogenase	43.3	89.2	9	0
LinJ.34.3030	Putative Alpha-keto-acid decarboxylase	39.2	72.3	33	0

* *Against the Human proteome*.

Thus, using the cross-reference between recovered and generated data, 31 proteins from *L. braziliensis* (Supplementary Material 5) and 37 proteins from *L. infantum* (Supplementary Material 6) were identified with evidence of being potential drug targets. Among these, 2 proteins in each proteome are important for network stability due to their highly connected neighborhoods. Seven and nine proteins, in *L. braziliensis* and *L. infantum*, respectively, are relatively essential in the network as they have a degree centrality >100. While 4 and 5 proteins have similarity value <50% and degree value > 50, based on known targets similar to these proteins, 1,105 and 145 compounds with the possibility of repurposingto be tested for their leishmanicidal potential, of which, only 9 compounds have already been tested against *Leishmania* (Supplementary Materials 5, 6).

## Discussion

Studies designed to select proteins for deep validation as drug targets are often carried out without assessing the whole proteome of a pathogen, and they are often focused on classic groups of proteins, neglecting new potentially druggable targets of therapeutic importance (Patel et al., 2013). The use of only traditional methods results in a lack of information about the real druggability of complete proteomes (Brown and Superti-Furga, 2003; Prinz et al., 2011). This fact becomes even more critical when talking about neglected diseases, for which the lack of information is not only concentrated on druggability, but it extends to other areas, such as the protein three-dimensional conformations and protein-drug interaction (Charlton et al., 2018). This reality turns out to be even more worrying considering the possibility of discovering new compounds by the pharmaceutical industry, since drugs for this group of diseases generate little financial return, thus reducing the efforts of this sector in financing research for the development of new drugs (Trouiller et al., 2002; Chatelain et al., 2013; Charlton et al., 2018). Thus, using approaches capable of analyzing complete proteomes searching for characteristics that can classify proteins as potential targets is an essential step that can help us answer questions about the druggable proteome.

In this way, we present here a modification of the methodology applied by Patel et al. (2013), capable of evaluating complete proteomes through the assessment of features about three-dimensional protein structure, druggability, functional class and subcellular location, overlapping these data with the gene expression profiles and protein topological information from interaction networks (Patel et al., 2013). In the analyses carried out by Patel et al. (2013) to identify druggable cancer proteins, the evaluation of such characteristics cited ealier was performed to select the most promising druggable proteins, however, these characteristics were not directly compared to drug targets, there was no evaluation of similarity semantics between GO terms, and structural alignments of the binding sites were not performed, being the only direct comparison carried out through sequence alignment between the cancer proteins and drug targets (Patel et al., 2013). The absence of these analyses might not have an impact on the results of Patel et al. (2013), since the authors from that work started their study with a very defined set of oncology proteins which could be considered as primary drug targets.

In contrast, our analyses started from complete proteomes without any predefined drug candidate, so it was necessary to apply an approach comparing protein by protein between the parasite's proteome and the BindingDB database, in order to identify potential targets. The significant difference between the approaches as well as the non-applicability of the approach carried out by Patel et al. (2013) for a complete proteome makes the results of both studies not directly comparable.

Therefore, we applied here an approach capable of evaluating the similarity of a proteome against known drug targets, considering the conformational and chemical similarity of the binding sites, the similarity of the molecular functions and biological processes those proteins are involved with, and their subcellular location. Furthermore, we integrated those data with their position in biological protein interaction networks, in order to provide a list of proteins that are important for the maintenance of the disease state and can be potential targets for drugs. This methodology also provides a list of compounds already developed with the ability to interact with the proteins selected as potential targets.

As cited in the results section, only three *L. infantum* proteins were assayed against drugs and their results are deposited in BindingDB: SIR2RP1(NAD-dependent protein deacetylase - LinJ.26.0200), CYP51 (Putative lanosterol 14-alpha-demethylase - LinJ.11.1100) and TRYR [N(1),N(8)-bis (glutathionyl) spermidine reductase - LinJ.05.0350]. SIR2RP1 has already been tested as a target for the compounds Nicotinamide and analogs, Surfactin, and Sirtinol, being described as target potential and essential for parasite survival (Vergnes et al., 2005; Zheng, 2013). This protein was not present in our results due to the absence of a predicted three-dimensional structure for it. However, it has significant similarity in terms of molecular function, biological process, and subcellular location with 234 known drug targets. CYP51 is a cytochrome 450 enzyme essential for steroidal biosynthesis that has enzyme activity inhibited by the compound VNI [(R)-N-(1-(2,4-dichlorophenyl)-2-(1H-imidazol-1-yl)ethyl)-4-(5-phenyl-1,3,4-oxadiazol-2-yl)benzamide)] and analogs (Friggeri et al., 2018). TRYR is one of the enzymes involved in the process of neutralizing reactive oxygen species, being a drug target already studied in trypanosomatids due to its essentiality for parasite survival and due to its significant difference from glutathione reductase, the equivalent enzyme in humans (Schmidt and Krauth-Siegel, 2005; Rodrigues et al., 2012). In pharmacological context, LinJ.05.0350 was described as a target for 1,3,4-thiadiazolium-2-aminide, a compound with antitumor, antibacterial, antifungal, and anti-inflammatory activity already described and that presents leishmanicidal activity inhibiting the growth of *Leishmania* species *in vitro* and resulting in the decrease of lesion size and parasitic load (Da Silva et al., 2002; Rodrigues et al., 2007, 2009, 2012).

LinJ.11.1100 and LinJ.05.0350 proteins were evaluated in our study with significant similarity for the 5 metrics used against known targets of BindingDB. In our results, LinJ.11.1100 was described as similar to Steroid 21-hydroxylase (P08686), Prostaglandin G/H synthase 2 (P79208), and Thromboxane-A synthase (P49430); making it possible to infer LinJ.11.1100 as a potential target for 49 compounds, among them the inhibitor of cytochrome 450 (2S,4S)-ketoconazole and the anti-inflammatory and anticancer resveratrol, which already had antipromastigote and antiamastigote effects described in *L. major, Leishmania amazonenesis* and *Leishmania donovani* (Kedzierski et al., 2007; Dinesh et al., 2014; Ferreira et al., 2014). LinJ.05.0350 protein, which has already been related to drug-resistant phenotype, was identified in our approach due to its similarity with Serine / threonine-protein kinase MRCK alpha (Q5VT25) and BRSK2 (Q8IWQ3), thus being inferred as a potential target for 28 compounds, including kinase inhibitors used as cancer treatment staurosporine and sunitinib, both compounds already described in the literature as potential anti-leishmanial (Dalton et al., 2010; Foucher et al., 2013; Sanderson et al., 2014; Balanco et al., 2019). Despite the high similarity with drug targets and the possibility of interaction with the compounds described above, LinJ.11.1100 and LinJ.05.0350 are not present in the final list of potential drug targets, since their genes did not show evidence of expression in the amastigote form (data on compounds with the possibility of interaction with these proteins can be retrieved from the Supplementary Material available at https://leishtargets.github.io/).

In addition to the three proteins of *L. infatum*, three other proteins of the genus *Leishmania* have the result of interaction with drugs deposited in the BindingDB, two of which belong to the proteome of *L. mexicana* and one of *L. tarentolae*. Orthologs for these proteins can be found in our results, however they are not present in the final list of potential drug targets for different reasons. The orthologous proteins of LmxM.34.4750 - Glyceraldehyde-3-phosphate dehydrogenase-like protein (LbrM.34.4710, LinJ.35.4810) were not recovered due to the absence of available three-dimensional structure. While the orthologs for LmjF.22.1360 - Farnesyl pyrophosphate synthase (LbrM.22.1240, LinJ.22.1210) and Q1A5Y2 - Tubulin alpha chain (LbrM.13.0200, LbrM.13.0190, LinJ.13.0330) were similar to BindingDB drug targets in the context chemical and biological, however they were not in accordance with criteria established in the methodology. This result allows us to assume that these proteins are potential drug targets, although it is not possible to infer interaction with the compounds covered in this study.

The multidisciplinary computational analysis allowed the identification of 68 proteins with the potential to be tested as new targets for the development or repurposing of drugs. Neither of these proteins has been described in BindingDB as a drug target. Evaluating the predicted GO terms for those 68 selected proteins, it was noticeable that we identified proteins related to functions already well-described and used by the pharmaceutical industry, such as those with kinase activity (Supplementary Material 1), a class of proteins that has already been used as drug targets for inflammatory, autoimmune diseases and cancer (Cohen, 2009; Cohen and Alessi, 2013; Patterson et al., 2014; Bhullar et al., 2018). This finding is consistent with the literature since that class of proteins has also been indicated as possible drug targets for the treatment of leishmaniasis caused by *L. donovani* and *Leishmania mexicana* (Ali et al., 2012; Rachidi et al., 2014; Catta-Preta and Mottram, 2018; Raj et al., 2019). The potential of neutralizing proteins of that class has also been demonstrated by the use of the kinase inhibitor Trametinib for amastigotes and promastigotes of *L. braziliensis* and *L infantum* (Borba et al., 2019). The same observation can be noted to targets with hydrolase activity and ATP-binding, classes of proteins that have been highlighted as potential targets (Cravatt and Lichtman, 2003; Chen et al., 2005; Soni et al., 2020), and also reported as a potential drug target in *L. infantum* (Chávez-Fumagalli et al., 2018; Sales et al., 2019).

Among the 68 potential targets it is possible to find proteins in both species that are potentially essential, but which are not currently widely explored as drug targets in *Leishmania* species, such as coproporphyrinogen III oxidase (LbrM.06.1260, LinJ.06.1330) and vacuolar protein sorting-associated protein 4 (LbrM.29.2470, LinJ.29.2610). This fact may be associated with the high similarity of these proteins with their orthologs in the human proteome. However, proteins such as small g-protein (LbrM.35.6570, LinJ.36.6510), which in our results were inferred as potential drug targets in both species, do not show high similarity with orthologs in humans, but remain unexplored as drug targets.

The applied methodology also allowed to identify proteins that until recently (in the version of the genome used in the study) were noted as hypothetical proteins, namely Major Facilitator Superfamily (LinJ.17.1550), Dephospho-CoA kinase (LinJ.18.0290), PPR repeat/Pentatricopeptide repeat domain/PPR repeat family (LinJ.36.5040), all with putative annotation; A fact that is repeated throughout the list, since among the 68 potential targets, 45 have putative annotation, thus showing that important proteins of the leishmanias druggable proteome still need further studies that reveal the full potential of these proteins to be exploited as targets drugs.

Combining the results of similarity matrices with gene expression and the protein interaction networks allowed the identification of 7 and 9 essential proteins for the amastigote evolutionary stage in *L. braziliensis* and *L. infantum*. Among these essential proteins it was possible to identify the putative DEAD box RNA helicase (LbrM.35.2040, LbrM.35.2370, and LinJ.36.2260) and putative ATP-dependent RNA helicase (LinJ.28.1420) proteins, which were reported as central by the average connectivity of neighbors, a fact that reflects their importance for parasite survival, since such proteins are essential for RNA metabolism, differentiation of amastigotes, and infectivity (Sharma and Jankowsky, 2014; Padmanabhan et al., 2016; Pandey et al., 2020). In our results, these proteins were similar to 10 drug targets of BindingDB according to the established criteria, thus we were able to infer the potential interactions, among these we had the already mentioned Staurosporine and Sunitinib, the protein kinase inhibitor Sorafenib and the antibiotic Novobiocin, both with antileishmanial activity already identified in experimental methods (Singh et al., 2005; Sanderson et al., 2014). Despite not having a low similarity with the human proteome, being highly conserved proteins, they can be inferred as potential drug targets, since when inactivated they result in an inability to differentiate into axenic amastigotes, loss of mitochondrial membrane potential, mitochondrial fragmentation, and cell death (Padmanabhan et al., 2016; Pandey et al., 2020).

The analysis of similarity against the human proteome allowed to verify the possibility of side effects, prioritizing the targets of *Leishmania* spp. most different from the human proteome. Cross-referencing these data with data on known targets allowed us to identify a list of compounds already developed with the possibility of interacting with potential selected targets. Among more than 1,000 compounds, there are only 15 compounds already tested for *Leishmania* species with assay described in BindingDB, considering all 68 targets selected, and only 9 compounds considering targets with low similarity to the human proteome. Evaluating the list of potential targets, it was possible to identify orthologs between the species covered in this work, showing the possibility of redirecting a drug with the potential to act in different parasite species. Among these compounds inferred as antileishmanial in our results are compounds already widely known, such as the general proteinase inhibitor Pepstatin A (BindingDB ID 912) and compounds poorly associated with a potential leishmanicide, such as C20H22N8O5 (BindingDB ID 18046), Gossypol (BindingDB ID 46555) and Bosutinibe chemotherapy (BindingDB ID 4552).

In both *Leishmania* species studied, GPDH proteins (LbrM.10.0640 and LinJ.10.0560) were identified as central proteins in the PIN and potential drug targets, a fact that is compatible with literature data (Suresh et al., 2000; Ogungbe and Setzer, 2013; Passalacqua et al., 2015) since such proteins are essential for parasite survival, and present structural characteristics consistent with the requirements for intelligent drug design (Cruz et al., 2012; da Silva et al., 2012; Belluti et al., 2014; Costa et al., 2020), being drug targets already studied in other trypanosomatids such as *Trypanosoma cruzi* (Leite et al., 2009; Maluf et al., 2013) and *Trypanosoma brucei* (Haanstra et al., 2017). In addition to having a key role in the glycolytic pathway that is related to the energy production of the parasite (Naderer et al., 2010; Zhao et al., 2018), those proteins participate indirectly in the pentose phosphate pathway, therefore they are related to the maintenance of the disease state, since that pathway represents an important process to parasitic defense against reactive oxygen species produced by hosts (Maugeri et al., 2003; Costa et al., 2020).

Costa et al. (2020) also reported GPDHs as potential drug targets describing regions of the proteins, which are essential for their molecular function in parasites, however, those segments are not conserved compared to mammalian species, highlighting the importance of these regions for drug development, a result that is also consistent with the data presented by Haanstra et al. (2017), who describes GPDH inhibitors in *T. brucei* that result in the death of trypanosomes without injury to host cells (Haanstra et al., 2017). In our results, those proteins were inferred as potential targets for more than 50 drugs, including Methotrexate, Pyrimethamine, and Quinoline compounds. Methotrexate shows an antileishmanial effect with a significant decrease in the average infection rate when associated with meglumine antimoniate (Mahmoudvand et al., 2017). However, the main use of this drug is currently related to the investigation of metabolic pathways in *Leishmania* species due to its inhibition function of folate metabolic pathways (Vickers and Beverley, 2011). The pyrimethamine has been evaluated for decades regarding its activity in leishmaniasis, and an antileishmanial effect has already been reported when in its combined forms (Neal, 1976; Serban, 2019). Quinoline compounds have already shown potent antileishmanial activity *in vitro* and *in vivo*, thus different formulations of these compounds have been tested as a treatment for leishmaniasis, due to their high efficiency when compared to the standard drug pentamidine, and due to the absence of toxicity to mammalian cells in the concentrations necessary for leishmanicidal action (Sahu et al., 2002; Tempone et al., 2005; Gopinath et al., 2014; Upadhyay et al., 2018).

Alcohol dehydrogenase proteins have been associated with miltefosine resistance in *Leishmania* parasites, as they are also involved with the reduction of reactive oxygen species and protection against oxidative stress (Carnielli et al., 2014; Hefnawy et al., 2017). This class of proteins has also been described as a potential pharmacological target in analyzes performed using the *T. cruzi* proteome (Alves-Ferreira et al., 2009) and in our analyzes, these proteins (LbrM.30.2040, LinJ.30.2100) were inferred as potential targets for formamide compounds, which are known to inhibit their activity (Gibbons and Hurley, 2004; Plapp, 2010; Espuelas et al., 2012), and targets for benzimidazole-based compounds, drugs for which leishmanicidal activity has been reported and used as antiparasitic agents that act in inhibiting tubulin polymerization (Mota et al., 2014; De Luca et al., 2018; Sánchez-Salgado et al., 2018).

Phosphoenolpyruvate carboxykinase protein (PEPCK) is described as a key factor in parasite gluconeogenesis, being expressed constitutively in both main evolutionary stages, and reported as a highly immunogenic antigen capable of triggering a robust T-cell-mediated immunity in humans (Rodriguez-Contreras and Hamilton, 2014; Mou et al., 2015). In our results, PEPCK (LinJ.27.1710) was identified as a potential target for Staurosporine compounds, protein kinase inhibitors also previously inferred with potential interaction for other proteins in the dataset of potential targets in *Leishmania* species, that have an effect on leishmaniasis preventing the growth of parasitic cells and reducing their virulence (Becker and Jaffe, 1997; Basmaciyan et al., 2018; Omura et al., 2018).

Although new analyses, both computational and using traditional methods, are necessary for the identification of targets and the development of new drugs, our study shows there are many druggable and pharmacological proteins capable of interacting with already developed compounds, and they can provide essential information for future studies to test the interaction between these targets and drugs, reducing the cost of developing new drugs and the time to discover new therapeutic approaches (DiMasi et al., 2003, 2010; Basak, 2012).

We present a modified approach to the methods developed by Patel et al. (2013) where new data has been integrated for the selection of potential targets. Thus, the methodology presented is a computational approach capable of selecting a list of proteins of pharmacological interest in a complete proteom, with evidence of interaction with drugs already available. It was also possible to provide a list of already available compounds with the ability to interact with the identified proteins. Thus, the application of this approach can be used as a first step in the intelligent selection of targets for drug repurposing, helping to prioritize proteins and compounds to be validated experimentally. Finally, we emphasize that the approach described here does not replace traditional methods capable of identifying details of the target/drug interaction, however, it can be used in conjunction allowing a wide evaluation of the possibilities within a proteome.

## Data Availability Statement

The datasets produced in this study can be found in Supplementary Materials and at the dedicated website: https://leishtargets.github.io/.

## Author Contributions

AR: conceptualization, project administration, resources, and supervision. CS: data curation and validation. CS and AR: formal analysis, methodology, writing – original draft, and writing – review and editing. All authors contributed to the article and approved the submitted version.

## Conflict of Interest

The authors declare that the research was conducted in the absence of any commercial or financial relationships that could be construed as a potential conflict of interest.
